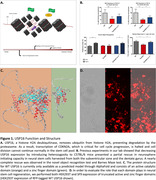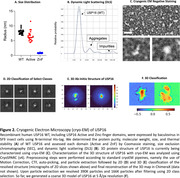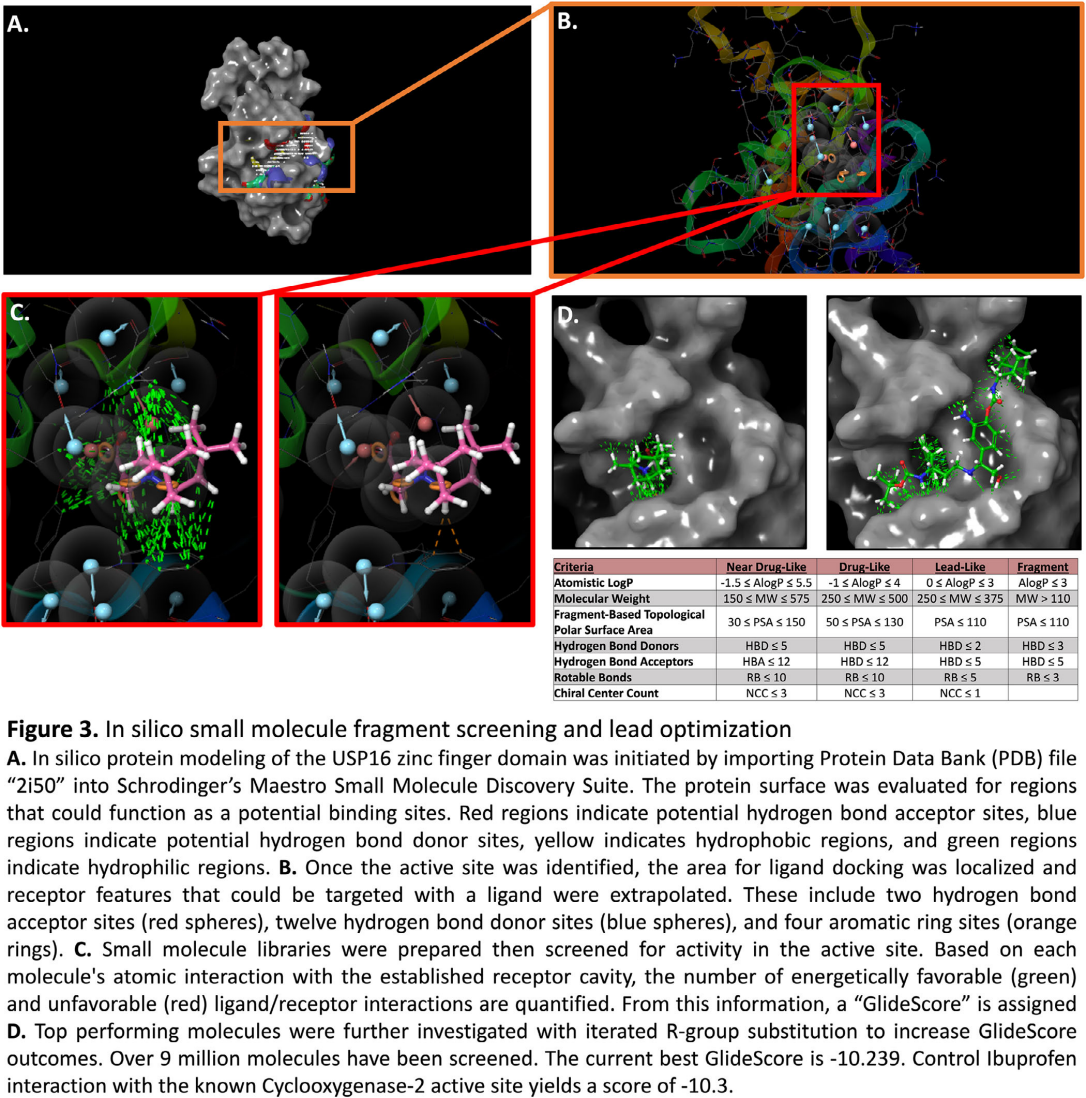# Identification of USP16 cryo‐EM protein structure and development of a small molecule inhibitor to restore compromised neural stem cell regeneration in a mouse Alzheimer’s model

**DOI:** 10.1002/alz.093300

**Published:** 2025-01-03

**Authors:** William Hai Dang Ho, Sabra I. Djomehri, Vincent M. Alford, Elizabeth Y. Chen, Felicia Reinitz, Sydney Gottlieb, Francie I. Igboabuchi, Grace Yuna Chung, Daniel Fernandez, Elizabeth Montabana, Bharti Singal, Zhen Qi, Angera H. Kuo, Dalong Qian, Michael F. Clarke

**Affiliations:** ^1^ Institute for Stem Cell Biology and Regenerative Medicine / Stanford University School of Medicine, Stanford, CA USA; ^2^ Macromolecular Structure Knowledge Center / Stanford University, Stanford, CA USA; ^3^ Cryo‐Electron Microscopy Center / Stanford University School of Medicine, Stanford, CA USA

## Abstract

**Background:**

Hallmark pathologies of Alzheimer’s Disease (AD) include the accumulation of both extracellular amyloid and intracellular tau proteins. While a significant body of knowledge exists surrounding the role of the protein aggregates in the context of AD, research supporting these as targets for therapeutic development have yielded inconsistent findings. One significant barrier is the inability to restore cognitive function despite the successful clearance of these proteins. In our recently published paper, we discuss a novel target for AD, USP16, which has been shown to promote ubiquitination of histones allowing for transcription of CDKN2A, a cell cycle regulator. We demonstrated that knockdown of USP16 improved neurosphere initiating capacity in mouse neural stem cells. Moreover, reduction of USP16 expression via heterozygosity resulted in improved cognition in 12‐month‐old mice. From these findings, our lab aims to identify the full‐length protein structure of USP16 using cryogenic electron microscopy (cryo‐EM) and develop a small molecule drug to inhibit its activity for end‐use as a viable AD therapeutic option.

**Methods:**

Truncated regions corresponding to the active and zinc‐finger (ZnF) domains of USP16 were expressed in both HEK293T and SF9 cells. Whole cell lysates were purified using affinity chromatography and identity was verified using Western blot. The purified protein products were submitted for cryo‐EM to determine their respective structures. Focusing on the ZnF domain, we utilized both a previously published proton NMR‐derived structure as well as AlphaFold‐predicted USP16 protein structure to serve as model scaffolding onto which we could perform in silico small molecule drug screening. Libraries were obtained from Schrodinger, MilliporeSigma, Emanine, MolPort, Thermo Fisher, MedChemExpress, Life Chemicals, ChemDiv, and Mcule.

**Results:**

Chromatogram from affinity chromatography and Western blot imaging together indicate successful isolation of truncated USP16 domains expressed in HEK293T and SF9 cells. Preliminary findings from cryo‐EM on the WT SF9 construct recapitulates the major domains predicted by AlphaFold. Over 9.5 million small molecule hit interactions against the ZnF domain have been identified.

**Conclusion:**

Cryo‐EM was successful in generating a course model of USP16. In silico protein modeling and drug screening was able to identify potential ligands against the ZnF domain of USP16.